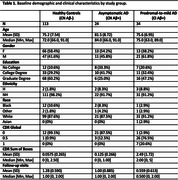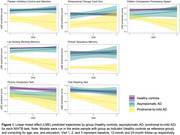# Computerized testing to detect cognitive decline in biomarker‐confirmed Alzheimer’s disease: Longitudinal findings from the ARMADA study

**DOI:** 10.1002/alz.094334

**Published:** 2025-01-09

**Authors:** Roos J Jutten, Emily Ho, Tatiana Karpouzian‐Rogers, Carol A. Van Hulle, Hiroko H Dodge, Cindy J. Nowinski, Richard C. Gershon, Sandra Weintraub, Dorene M Rentz

**Affiliations:** ^1^ Massachusetts General Hospital, Harvard Medical School, Boston, MA USA; ^2^ Northwestern University Feinberg School of Medicine, Chicago, IL USA; ^3^ Wisconsin Alzheimer’s Disease Research Center, University of Wisconsin School of Medicine and Public Health, Madison, WI USA

## Abstract

**Background:**

The National Institutes of Health Toolbox for Assessment of Neurological and Behavioral Function (NIHTB) was developed to address the need for a brief yet comprehensive instrument to facilitate more uniform assessment in large‐scale research studies. Here, we investigated whether the cognitive measures of the NIHTB detect cognitive decline in biomarker‐confirmed Alzheimer’s disease (AD).

**Method:**

We used data from N = 178 participants (age 76.5±8; 53% female) from the Assessing Reliable Measurement in Alzheimer’s Disease and Cognitive Aging (ARMADA) study, who were recruited across nine research centers in the United States and had amyloid PET or amyloid‐beta42/tau CSF data available within two years of their baseline NIHTB assessment. Participants' cognitive status (normal (CN) or impaired (CI), based on standardized protocols) and amyloid status (Aß‐/Aß+, based on site‐specific cut‐offs) were combined to create three groups: healthy controls (CN Aß‐, n = 113), asymptomatic AD (CN Aß+, n = 24), and prodromal‐to‐mild AD (CI Aß+, n = 34) (Table 1). The NIHTB was assessed at baseline, 12 and 24 months and includes five tasks measuring fluid cognition (attention, executive functions, processing speed, working memory, and episodic memory) and two tasks reflecting crystallized cognition (oral word reading and auditory word comprehension). Linear mixed‐effect models correcting for age, sex, and education were used to examine cross‐sectional and longitudinal NIHTB scores in the three study groups.

**Result:**

Compared to healthy controls, the prodromal‐to‐mild AD group had lower baseline scores on most NIHTB fluid tasks (Flanker Inhibitory Control and Attention, Dimensional Change Card Sort, Picture Sequence Memory, List Sort Working Memory) and showed accelerated decline over 12‐24 months on Pattern Comparison Processing Speed, List Sorting Working Memory and Picture Vocabulary (Figure 1). None of the NIHTB tasks detected cross‐sectional or longitudinal differences in the asymptomatic Aß+ group compared to healthy controls over the course of this study.

**Conclusion:**

The NIHTB detects cross‐sectional impairment in episodic memory, attention, executive functions and working memory and longitudinal change in processing speed, working memory and auditory word comprehension in prodromal‐to‐mild AD. These findings further support the use of the NIHTB in AD research, but also imply that the optimal NIHTB composite to detect cognitive decline may differ across AD clinical stages.